# Light Dosing and Tissue Penetration: It Is Complicated

**DOI:** 10.1089/photob.2020.4843

**Published:** 2020-07-14

**Authors:** Raymond Lanzafame

**Affiliations:** Raymond J. Lanzafame, MD PLLC, Rochester, New York, USA.

Standing in a darkened room or being in the woods at night with a flashlight pressed against a hand or face to scare an unsuspecting sibling or confrere entering the space or just to marvel at the eerie red glow of light passing through the tissue was arguably a fond memory of childhood. This no doubt would date me if I were to disclose that the flashlight was usually a cheap battery-operated version that was powering a small incandescent bulb. The salient point is that this simple childhood practice demonstrated that a low power noncoherent beam of light could be transmitted through tissues in sufficient quantities to be visible to the naked eye.

This simple real-world observation arguably flies in the face of decades of arguments and a large number of experiments and publications presented as evidence to support or refute the utility of light-emitting diodes (LEDs), that is, lasers, laser diodes, and other light sources for photobiomodulation (PBM) or other applications. Some of these positions have been promulgated by industry in its attempt to differentiate one's product as having attributes that make it superior to various competitors for various reasons. Coherence, versus lack thereof, and so called high intensity and treatises on “Laser versus LED” and other variations have muddied understanding of the fact that when the proper wavelengths of light are applied to an appropriate and receptive tissue at the proper parameters, the observed effects of photobiomodulation therapy (PBMT) accrue.^1^

These issues are further compounded by the selection and use of light wavelengths for PBMT based on their “tissue penetration depth” primarily rather than specifically matching wavelengths with the spectral absorption curves of the target chromophores. This topic is worthy fodder for another editorial in and of itself since we tend to oversimplify complex biological processes by attempting to explain them based on light interaction with a handful of potential photoacceptors. That fact notwithstanding, the heuristic that states that longer wavelengths penetrate tissue more deeply has also been shown to be the case.

Being able to target tissues at depth is an important concern regardless of whether one is attempting PBMT or a photocoagulation or photoablative event. One still needs to get the right amount of energy to the specific target. The time course over which this is delivered is also important. Mathematical reciprocity of exposure time and irradiance to achieve a specific light dose can be ineffective or be deleterious.^2,3^ Some have argued that all cells and tissues should be responsive to PBMT if the proper wavelengths and dosing parameters are delivered, particularly since mitochondria and cytochrome c oxidase, a primary PBM target photoacceptor, exist in eukaryotic cells. However, it is clear that some cells are “bystanders,” being affected or not based on their proximity to their neighbors in the milieu, or only after other cells produce the requisite cytokines or substrates necessary to kickstart their cellular machinery.^4^

This brings us back to the issue of delivering light to the target tissue. There have been a number of studies and treatises on depth of penetration of light in tissues over the years. Many have used point sources or focused beams and measured light transmission with various methodologies. There is often an underlying assumption that a more focused higher power incident beam results in better delivery of light at depth. However, Keijzer et al. demonstrated >30 years ago that a specific wavelength of light delivered to tissue at a uniform and constant power density resulted in substantially greater depths of penetration when the light was delivered over a larger area (spot).^5^ Two other features of note in their study was the fact that the irradiance required to achieve the same power density over the larger area was significantly greater than that for the small spot, and they also found that laser light focused on tissue behaved in the same manner as the same wavelength delivered through a fiber.^5^ Therefore, it would make sense to use light arrays or larger diameter beams with sufficiently high irradiances for PBMT regimes rather than treating using multiple treatments at individual points, at least for some PBM applications.

Hu et al. added gender as yet another variable affecting the penetration of light in tissue.^6^ They studied the depth of penetration of 660 nm light delivered through an LED array in an effort to determine the effects of irradiance, tissue thickness, skin tone, gender, and bone and muscle content in both live human and cadaveric tissues for a 15–500 mW/cm^2^ range of irradiances. They found that light penetration was unaffected by skin tone, increased with irradiance and relative bone/muscle composition, and decreased with greater tissue thickness and in males. Tissue penetration depths were greater for females than for males.^6^ Live and cadaveric tissue penetration did not differ statistically for tissues <50 mm but cadavers required more red light to penetrate >50 mm.^6^ They also found like Keijzer et al. that although 100 mW/cm^2^ could penetrate <50 mm thick tissues, “a disproportionate irradiance increase” was required to achieve penetration depths >50 mm.^6^ The authors do point out that their studies should be carefully undertaken at different wavelengths and with different tissues since variations in melanin concentrations and tissue water can have different effects.^6^ This is prudent advice indeed.

It is necessary to take a number of variables into consideration and to understand that the wavelength, the intended target, and the method of light delivery are of great importance. The gender of the patient, although often neglected, may well also affect results and may partially explain variability in published study results and our own clinical outcomes. The use of light arrays or larger beams of light is emerging as strategies that can increase the likelihood of getting the desired light energy to the desired target. Careful attention to parameters is critically important. Despite our innate desire to simplify, there is no singular or simple answer for light dosing and tissue penetration to achieve optimal outcomes for PBMT regimes.
